# Cancer Hallmarks Analytics Tool (CHAT): a text mining approach to organize and evaluate scientific literature on cancer

**DOI:** 10.1093/bioinformatics/btx454

**Published:** 2017-07-14

**Authors:** Simon Baker, Imran Ali, Ilona Silins, Sampo Pyysalo, Yufan Guo, Johan Högberg, Ulla Stenius, Anna Korhonen

**Affiliations:** 1 Computer Laboratory, University of Cambridge, Cambridge, UK; 2 Language Technology Lab, Department of Theoretical and Applied Linguistics, University of Cambridge, Cambridge, UK; 3 Institute of Environmental Medicine, Karolinska Institutet, Stockholm, Sweden

## Abstract

**Motivation:**

To understand the molecular mechanisms involved in cancer development, significant efforts are being invested in cancer research. This has resulted in millions of scientific articles. An efficient and thorough review of the existing literature is crucially important to drive new research. This time-demanding task can be supported by emerging computational approaches based on text mining which offer a great opportunity to organize and retrieve the desired information efficiently from sizable databases. One way to organize existing knowledge on cancer is to utilize the widely accepted framework of the Hallmarks of Cancer. These hallmarks refer to the alterations in cell behaviour that characterize the cancer cell.

**Results:**

We created an extensive Hallmarks of Cancer taxonomy and developed automatic text mining methodology and a tool (CHAT) capable of retrieving and organizing millions of cancer-related references from PubMed into the taxonomy. The efficiency and accuracy of the tool was evaluated intrinsically as well as extrinsically by case studies. The correlations identified by the tool show that it offers a great potential to organize and correctly classify cancer-related literature. Furthermore, the tool can be useful, for example, in identifying hallmarks associated with extrinsic factors, biomarkers and therapeutics targets.

**Availability and implementation:**

CHAT can be accessed at: http://chat.lionproject.net. The corpus of hallmark-annotated PubMed abstracts and the software are available at: http://chat.lionproject.net/about

**Supplementary information:**

Supplementary data are available at *Bioinformatics* online.

## 1 Introduction

Cancer is the leading cause of mortality and morbidity worldwide according to the International Agency for Research on Cancer (IARC, 2014). Cancer researchers have recently evaluated the complexity of cancer and discussed the risk factors (intrinsic versus extrinsic) that may contribute to the development and promotion of the disease (Tomasetti and Vogelstein, 2015; Wu *et al.*, 2016). Although cancer research has developed greatly in recent past, further advances in this area will depend significantly on better understanding of the Hallmarks of Cancer and associated molecular pathways underpinning the mechanisms involved (Hanahan and Weinberg, 2011). While scientific literature is the most reliable and comprehensive source of knowledge to drive new research, its exponential growth in recent years is the bottleneck to extracting cancer-relevant information from existing literature. To support this time-demanding task, there is a need to develop a tool that can identify and extract the information critically needed, for instance, for cancer diagnostics, treatment and prevention.

Text Mining (TM) technology provides a solution for bridging the knowledge gap between free-text and structured representation of related information in cancer research (Spasic *et al.*, 2014). TM uses computational techniques such as Natural Language Processing (NLP) to automatically retrieve, extract and discover novel information in large databases. It can help humans to identify and verify required information from text more efficiently and it can uncover information or connections obscured by the huge volume of available literature. A number of TM solutions have been developed to support research in biomedicine, many of which are also applicable to cancer research (for a relatively recent review see e.g. (Zhu *et al.*, 2013)).

An important aspect currently not captured sufficiently by existing TM tools is the Hallmarks of Cancer (HoC). Introduced by Hanahan and Weinberg (2000, 2011), this framework is based on the idea that normal cells require certain characteristics (i.e. hallmarks) to behave as malignant cells. Proposed as a strategy to capture the complexity of cancer in a few basic principles, it provides an organized framework comprising of ten hallmarks. In Baker *et al.* (2016b), we took the first step towards identification of HoC in scientific literature. We introduced a supervised Machine Learning (ML) approach capable of classifying PubMed abstracts by the ten cancer hallmarks. Our evaluation showed that the resulting semantic classification was reasonably accurate.

In this paper, we present a novel Cancer Hallmarks Analytics Tool (CHAT). This end-user tool utilizes improved methodology to classify relevant literature according to a detailed and extensive cancer hallmarks taxonomy, designed to support the process of literature review in the field of cancer research, CHAT works on a large scale: it classifies over 150 million sentences extracted from over 24 million PubMed abstracts.

The extended taxonomy integrated in CHAT comprises not only the ten principal classes in the original HoC classification but also twenty-seven subclasses, representing the most important cellular processes involved in cancer development and promotion under the framework (Hanahan and Weinberg, 2011). Each hallmark class can be associated with several keywords and phrases which, when found in literature, represent good indicators for the presence of the hallmarks in text. Cancer researchers use systems such as PubMed for keyword-based queries. However, due to the scope and complexity of cancer literature, the number of keywords, their synonyms and possible combinations exceeds what researchers can memorize and manage. Also, overly complex queries can fail to achieve a satisfactory level of precision and recall. Our automatic classification approach captures the combinations and correlations of such keywords, along with other semantic information and metadata, which are input into the ML algorithm as features.

Our improved approach for hallmark classification uses methodology designed for more detailed, sentence-level classification, whereas the previous approach in Baker *et al.* (2016b) classifies only on an abstract-level. The NLP pipeline for sentence-level classification utilizes a new set of features and tools, as well as a new sentence-level annotated corpus. These resources are made publicly available under open licences as part of this paper.

We present direct evaluation of the methodology along with case studies that focus on lung and colorectal cancer, chemotherapeutic drugs as well as the growth factors that are relevant in cancer development. Our evaluation shows that CHAT automatically organizes and classifies the literature with good accuracy, and identifies the key correlations which are in line with the existing knowledge. Developed in close collaboration with cancer researchers, CHAT can be of great use for classifying scientific literature by cancer hallmarks and associated biological processes.

## 2 Materials and methods

The key components of the taxonomy including the principles of taxonomy creation (the Hallmarks of Cancer), the annotated corpus of PubMed abstracts, and the ML classifiers are described in the following subsections.

### 2.1 Taxonomy development

**Fig. 1.**
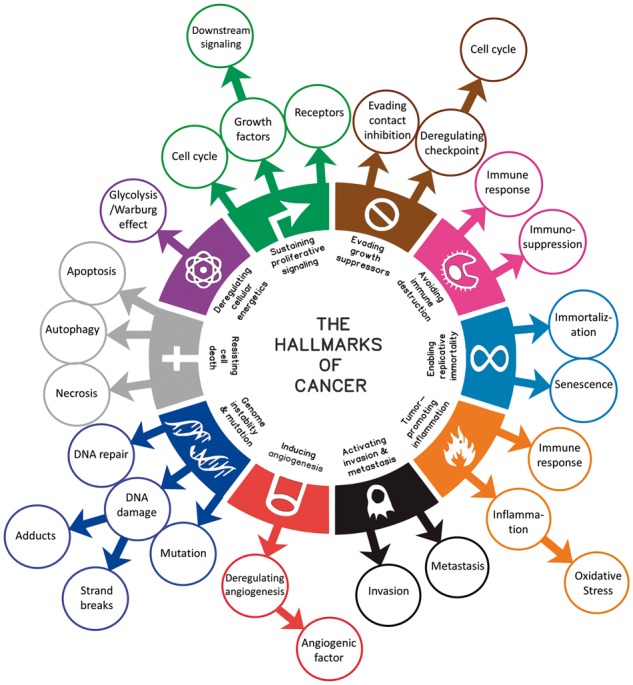
The *Hallmarks of Cancer* taxonomy. The inner circle represents the main ten cancer hallmarks and the outer circles indicate the cellular processes associated with each cancer hallmark as described in (Hanahan and Weinberg, 2011)

We extend and refine the original ten HoC by adding subclasses representing different biological processes linked to each hallmark as described in (Hanahan and Weinberg, 2011). The extended taxonomy consists of two levels: the first level contains ten primary classes representing the main cancer hallmarks and the second level consists of subclasses that represent more specific cellular or molecular processes. The overall taxonomy contains 37 classes (illustrated in Fig. 1).

### 2.2 Corpus annotation

By using search terms associated with each hallmark, literature collected from PubMed with a previously described strategy (Baker *et al.*, 2016b) was annotated to create a corpus for ML. In addition, search terms suitable for gathering a larger, representative sample of the literature for each hallmark and subcategories were used. Unlike in previous work, annotation was also carried out on a sentence level. Such annotation was conducted when clear evidence for, or meaningful association with, one or several hallmarks was found. It was performed for at least PubMed 200 abstracts per hallmark category (including also the subcategories). In case of obscurity, the context of the whole abstract was considered when deciding the relevance to certain hallmark(s), and for some hallmarks additional annotation was conducted to increase the performance. Sentences were annotated with hallmarks only when there was explicit evidence of association, such as the presence of keywords or phrases. Table 1 shows examples of sentences and keywords indicated as evidence for the corresponding hallmark annotation.

**Table 1.** Examples of sentences and keywords as evidence for annotated hallmarks

Annotated hallmark	Examples of sentences with evidence (highlighted) for the annotated hallmarks
Sustaining proliferative signalling—cell cycle	Results indicate the PCNA labelling with PC10 is a simple method for assessing the proliferative activity in formalin-fixed, paraffin-embedded tissue of NSCLC and correlates well with Ki-67 labeling and S-phase fraction of the cell cycle.
Evading growth suppressors—cell cycle check points & contact inhibition	Subsequently, sod3-transduced MEF cells developed co-operative p21-p16 downregulation and acquired transformed cell characteristics such as increased telomerase activity, loss of contact inhibition, growth in low-nutrient conditions and in vivo tumorigenesis.
By deregulating angiogenesis—angiogenic factors	Phosphorylated Akt and VEGF-A are involved in angiogenesis of gastric adenocarcinoma, and Akt activation may contribute to angiogenesis via VEGF-A upregulation.
Genomic instability and mutations—DNA repair	Incubation of BLM-treated cells dCF/dAdo resulted in significant inhibition of the repair of BLM-induced DNA SSB.
Activating invasion and metastasis—metastasis	Occurrences of metastases during γ-IR treatment accompanied induction of EMT markers, including increased MMP activity.

**Table 2.** Summary data and performance statistics for each class in the HoC taxonomy, where the **# Annotated** column is the number of positively annotated sentences in our training corpus, **# Classified** is the number of sentences in PubMed positively classified by our classifiers and **# Features** is the total number of features used by our classifiers

Hallmark	# Annotated	# Classified	# Features	Precision (%)	Recall (%)	F1-score (%)	Accuracy (%)
1. Sustaining proliferative signalling	993	811,719	7479	36.5	67.1	47.3	91.5
1.1 Cell cycle	320	141,941	3631	48.5	60.3	53.8	98.1
1.2 Growth factors growth promoting signals	323	224,980	3407	27.0	35.3	30.6	97.0
1.2.1 Downstream signalling	138	69,880	1952	41.2	29.0	34.0	99.1
1.3 Receptors	345	278,561	3558	33.3	54.5	41.4	96.9
2. Evading growth suppressors	366	579,810	4237	39.0	62.0	47.9	97.2
2.1 By deregulating cell cycle checkpoints	251	144,562	2908	32.9	49.4	39.5	97.8
2.1.1 Cell cycle	238	139,071	2747	33.6	46.6	39.1	98.0
2.1 By evading contact inhibition	118	273,566	1864	68.5	83.1	75.1	99.6
3. Resisting cell death	832	863,918	7141	56.5	82.1	66.9	96.1
3.1 Apoptosis	610	594,979	5841	60.7	79.8	69.0	97.5
3.2 Autophagy	157	33,845	1098	61.4	79.0	69.1	99.4
3.3 Necrosis	108	198,429	1682	66.9	76.9	71.6	99.6
4. Enabling replicative immortality	295	49,223	2323	59.0	85.8	69.9	98.8
4.1 Immortalization	111	6,407	1193	61.7	73.9	67.2	99.5
4.2 Senescence	185	39,298	1620	62.8	85.9	72.6	99.3
5. Inducing angiogenesis	358	308,574	2854	40.2	66.2	50.0	97.3
5.1 By deregulating angiogenesis	350	287,854	2776	40.3	65.4	49.9	97.4
5.1.1 Angiogenic factors	171	118,377	1696	42.5	53.2	47.3	98.8
6. Activating invasion and metastasis	667	943,054	5218	54.5	75.9	63.4	96.7
6.1 Invasion	282	271,211	3202	50.1	62.4	55.6	98.4
6.2 Metastasis	317	591,214	3383	53.8	71.3	61.3	98.4
7. Genomic instability and mutation	768	1,397,318	5675	36.3	72.7	48.4	93.2
7.1 DNA damage	371	193,566	3522	39.2	70.9	50.5	97.0
7.1.1 Adducts	97	37,599	918	59.2	62.9	61.0	99.6
7.1.2 Strand breaks	121	30,174	1515	32.9	47.1	38.8	99.0
7.2 DNA repair mechanisms	213	95,510	2483	39.2	61.0	47.7	98.4
7.3 Mutation	215	826,072	2042	36.8	61.4	46.0	98.2
8. Tumor promoting inflammation	518	1,145,524	4659	40.1	66.6	50.1	96.1
8.1 Immune response	78	117,320	1017	25.0	34.6	29.0	99.2
8.2 Inflammation	452	928,736	4445	42.4	66.8	51.8	96.8
8.2.2 Oxidative stress	241	220,979	2605	46.1	61.4	52.7	98.5
9. Cellular energetics	213	84,204	2006	45.8	79.8	58.2	98.6
9.1 Glycolysis/Warburg effect	195	48,772	1870	47.1	74.9	57.8	98.8
10. Avoiding immune destruction	226	651,044	2237	32.2	59.3	41.7	97.9
10.1 Immune response	152	465,785	1696	23.2	38.2	28.9	98.4
10.2 Immunosuppression	70	70,881	1035	51.5	50.0	50.7	99.6
			Macro-average:	45.1	63.6	52.3	97.9
			Micro-average:	43.7	66.8	52.8	97.9

The annotation was performed by an expert with over 15 years of experience in cancer research. The XML-based annotation tool described in (Guo *et al.*, 2012) was used, with some of its features adapted to the hallmark task.

**Fig. 2.**
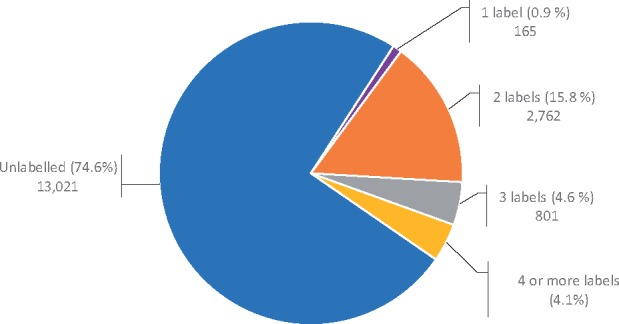
The distribution of the number of labels per sentence in the annotated corpus

About 75% of the sentences in the corpus are not labelled with a relevant hallmark (as shown Fig. 2). Most of the labelled hallmarks are associated with two hallmark labels (16.8%), typically due to a hypernymy relationship between the subclasses in the taxonomy, while only 0.9% of the sentences are labelled with exactly one hallmark label (i.e. with exactly one of the ten top-level classes).

To investigate the accuracy of annotations, we performed inter-annotator agreement analysis where a second expert annotator was asked to annotate a subset of 4963 sentences which were compared to those of the original annotator. We calculated the inter-annotator agreement using Cohen's Kappa (κ). We found an agreement of κ = 0.67 for the ten hallmarks, and κ = 0.61 for the entire taxonomy, indicating a substantial level of agreement among our experts (Fleiss *et al.*, 2013; Landis and Koch, 1977).

### 2.3 Natural language processing

**Fig. 3.**
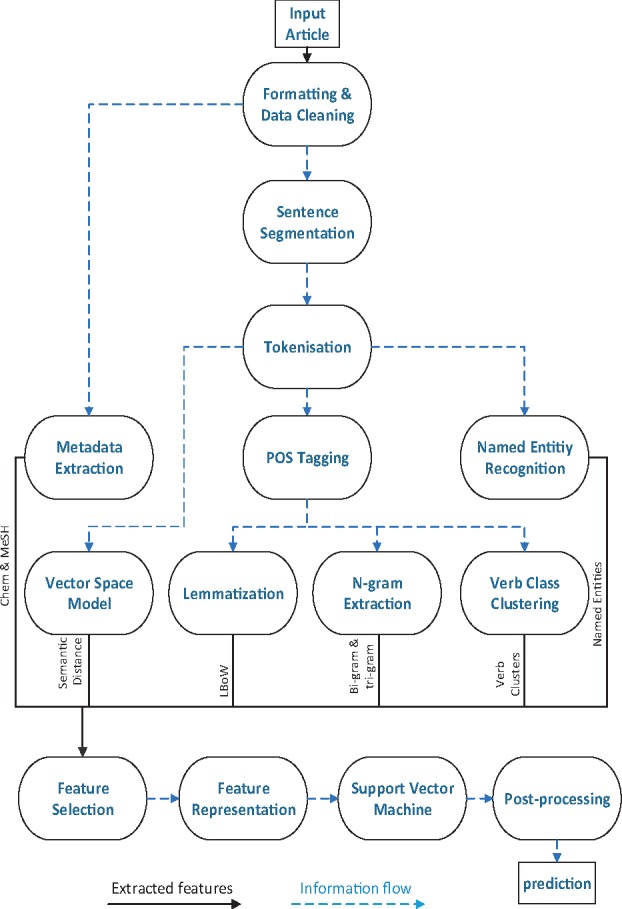
An illustration of the NLP pipeline used in CHAT

We designed and implemented a supervised NLP pipeline (Fig. 3) that extracts seven types of semantic and syntactic features from scientific literature:

**Lemmatized Bag of Words (LBoW):** The simplest feature employs all the words occurring in input texts. We lemmatize the words to reduce feature sparsity.

**N-grams:** We use standard bigrams and trigrams of words occurring in the input text.

**Verb classes (VC):** Verb classes group semantically similar predicates together, providing the means to abstract away from individual verbs when faced with data sparsity. We use the hierarchical classification of 399 verbs by Sun and Korhonen (2009) which was automatically acquired from cancer risk assessment literature using clustering.

**Named entities (NE):** Named entities capture domain specific concepts in texts, providing another way to group words into meaningful categories. We use five named entity types which are particularly relevant to cancer research: Proteins, DNA, RNA, Cell line and Cell type.

**Medical Subject Headings (MeSH):** A comprehensive controlled vocabulary for indexing journal articles and books in the life sciences. Most abstracts in our dataset contain an associated list of MeSH terms which we employ as features.

**Chemical lists (Chem):** Hallmark-related processes may involve chemicals. Since most abstracts in our corpus also contain a list of associated chemicals as metadata, we use these as features.

**Semantic distance (SD):** We construct a semantic vector space model (VSM) to capture the semantic similarity between words that appear in the corpus, and the hallmark labels. We use the approach presented in (Baker *et al.*, 2016a), where we train an artificial neural network (ANN) model that learns an embedded representation of words and labels jointly. We feed the ANN sentences and corresponding hallmark labels; the ANN creates a vector space where each (non-stop) word and hallmark label are presented as points (i.e. an embedded representation). We then use cosine similarity to measure the distance between words occurring in the sentence and a given hallmark label.

We use the GENIA tagger (Kulick *et al.*, 2004; Tsuruoka and Tsujii, 2005; Tsuruoka *et al.*, 2005) to perform the POS tagging, lemmatization and named entity recognition steps of the pipeline. The MeSH and Chem features are extracted from metadata provided by PubMed. These are associated with abstracts and not sentences (unlike the other features used), however, they still provide information that is beneficial for sentence classification. We associate this metadata with every sentence in the abstract.

We apply feature selection: features that are deemed too rare or too common in the annotated corpus are filtered out, so that only the most discriminating ones are used. The thresholds are set for each of the hallmarks by a process of trial and error, typically a minimum threshold value of five occurrences, while the maximum threshold varies greatly depending on the feature type (usually a value greater than 500 occurrences). This improves accuracy and reduces training time. This procedure is done separately for each of the hallmarks, i.e. we only select the features in the corpus that occur in abstracts annotated with the given hallmark. Therefore, each classifier has a unique set of selected features. The number of features for each hallmark after feature selection is given in Table 2; we also provide in the Supplementary Material the breakdown of the number of selected features for each feature type in Supplementary Table S1.

The features are represented in a sparse binary format for each sentence, with a value of ‘1’ indicating that the given sentence contains this feature.

The binary features are then input into 37 classifiers (support vector machines with linear kernels) that label each sentence with a binary label indicating its relevance to one of the 37 labels in the hallmark taxonomy. Each of the classifiers is trained and executed independently to allow for mutually non-exclusive multi-label classification. We use One-vs-Rest (OVR) training scheme, where each classifier is trained on the entire corpus. Sentences annotated with a hallmark label are counted as positive examples for training that classifier; otherwise, they are considered negative examples.

We use the hypernym/hyponym relationships in our taxonomy to determine whether an example should be labelled positively or negatively for a given hallmark node label, i.e. we consider subclass labels as positive examples when we are classifying their parent nodes. For example, when classifying the hallmark ‘resisting cell death’, the sentences annotated with the subclass ‘apoptosis’ would be considered positive examples for ‘resisting cell death’. Since we have heavily imbalanced classes (far more negative examples than positive ones), we apply inverse proportional class weighting to adjust for this imbalance.

We use Scikit-learn (Pedregosa *et al.*, 2011) to implement the SVM classifier step of the pipeline. The post-processing step at the end of the pipeline integrates the predictions of the individual 37 independent binary classifiers into a coherent form: if there is disagreement between a child node and its parent; one can either favour the child node’s prediction or the parent’s. We tested both strategies empirically and found that the latter alternative results in higher performance overall. This perhaps is expected with the data, since the leaf (child) nodes have fewer labelled examples in the corpus, and therefore on average would have weaker classifiers.

### 2.4 User interface

**Fig. 4.**
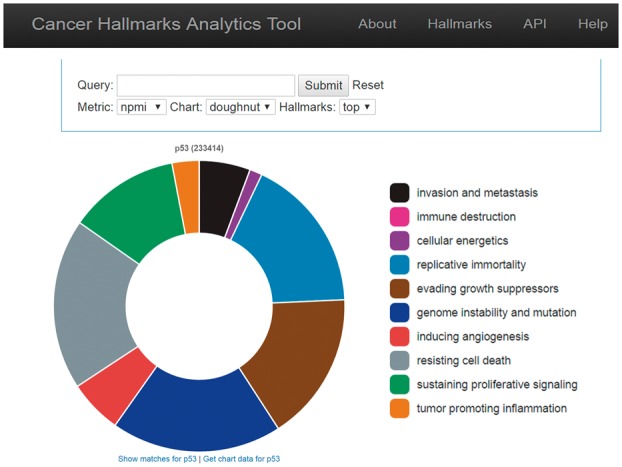
CHAT visualizes the hallmarks distribution for an input query (in this example, ‘p53’). There are several visualization options; in this example, the hallmarks are depicted in a ring akin to the original Hallmarks of Cancer publication (Hanahan and Weinberg, 2000)

**Fig. 5.**
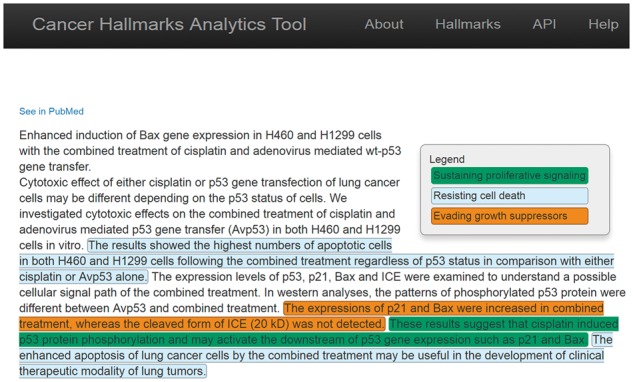
CHAT allows the user to explore individual abstracts, and visualizes the hallmark labels appearing in the text

**Fig. 6.**
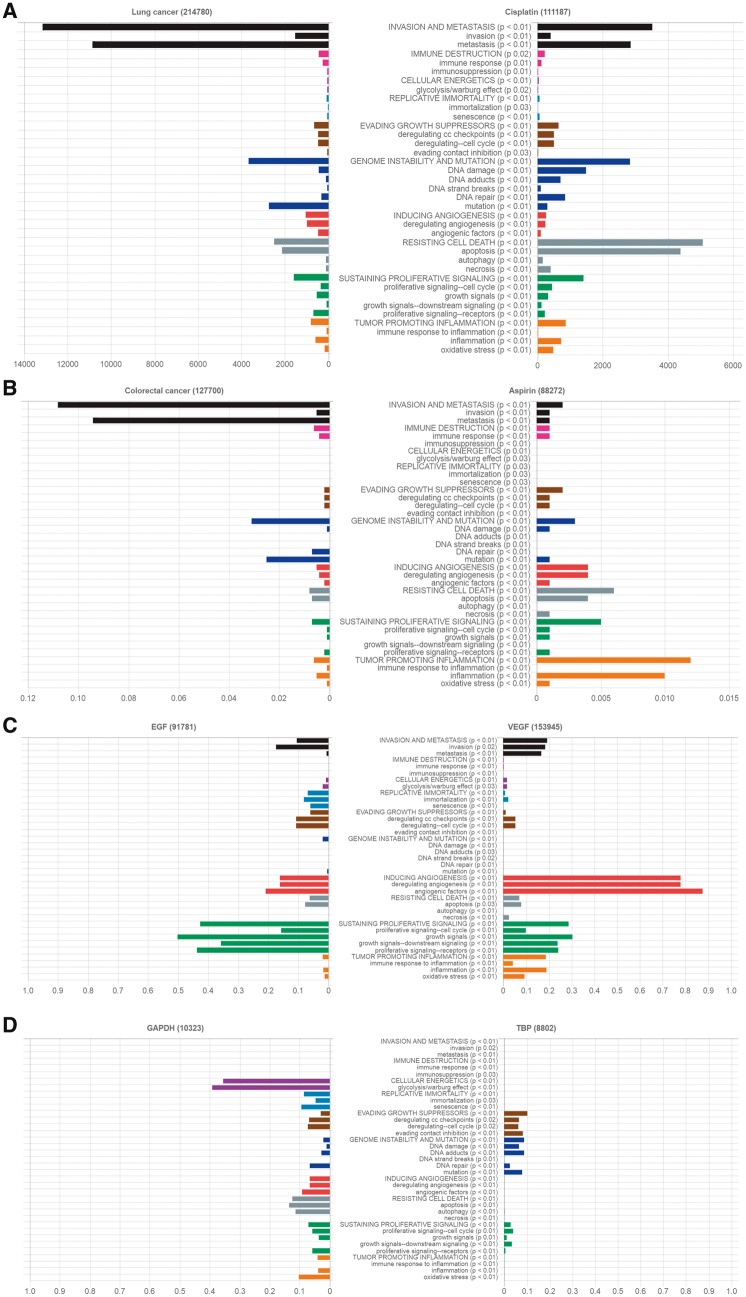
Automatic CHAT classification of the PubMed literature according to HoC taxonomy. Literature profiles; (**A**) lung cancer and cisplatin (data shown as Raw counts), (**B**) Colorectal cancer and Aspirin (data shown as CPROB; conditional probability), (**C**) growth factors EGF and VEGF (data shown as NPMI; normalized pointwise mutual information) and (**D**) housekeeping genes GAPDH and TBP (data shown as NPMI). Each bar represents the association for a cancer hallmark and/or associated biological process with the search query. The p-value is based on either Fisher-exact test or Chi-squared test followed by a Bonferroni correction

We integrated PubMed documents and the hallmark sentence classification generated by our NLP pipeline into a database, and in close consultation with cancer researchers, created a web-based interface (Figs 4–6) that allows users to analyze the distribution of a search query of interest with respect to the hallmarks using multiple visualizations. Several options are provided for user metrics: raw counts, conditional probability values (i.e. the probability of the sentence being assigned the hallmarks given the query), Point-wise Mutual Information (PMI), and normalized PMI (NPMI), which are calculated as follows:

$$P\left(h||q\right)=\frac{P(h,q)}{P(q)}\;PMI=log\left(\frac{P(h,q)}{P(h)P(q)}\right)\;NPMI=\frac{PMI\left(h,q\right)}{-log(P(h,q))}$$

where h and q denote a given hallmark and a search query. The UI enables the user to explore the source data and to assess the evidence for specific associations between query terms and the hallmarks (Fig. 5). In addition, the UI allows the user to compare two queries on the same graph (Mirrored bar graph) as well examine the statistical significance results of the comparison (illustrated in Fig. 6).

In the comparison screen, the tool automatically employs as a statistical test either the Fisher-exact test or Chi-squared test followed by a Bonferroni correction. We use the Fisher-exact test if the expected frequency is less than five as typically recommended by statisticians (McDonald, 2009). CHAT also allows the user to download the data displayed in the graph for further analysis.

In terms of implementation, we first indexed all of PubMed (2016 release) and the generated hallmark label prediction by our NLP pipeline using Lucene (https://lucene.apache.org), a state-of-the-art indexing and text search engine. We then created a web interface using the Python Flask framework (http://flask.pocoo.org) to allow flexible querying of the data, and implemented client-side visualization of results using the Chart.js Javascript charting library (http://www.chartjs.org). We plan to update our indexed articles annually for every PubMed release.

## 3 Results

We first describe the intrinsic evaluation of the NLP pipeline (i.e. hallmark classifiers) using standard methods and metrics. We then assess the functionality and the practical usefulness of CHAT with several case studies on cancer research.

### 3.1 Intrinsic evaluation

We evaluate the performance of the trained classifiers intrinsically against the annotated gold-standard dataset using standard performance measures:

$$Precision=\;\frac{TP}{TP+FP}Recall=\;\frac{TP}{TP+FN}$$

$$Accuracy=\;\frac{TP+TN}{total}F1\;score=2\frac{precision\times recall}{precision+recall}$$

where TP, FP, TN, FN are the True Positives, False Positives, True Negatives and False Negatives respectively. We use nested cross-validation to avoid sampling bias, as recommended for small datasets (Statnikov *et al.*, 2008; Varma and Simon, 2006). The data is divided into four folds, i.e. the model is trained with 75% of the data and tested with the remaining 25%, and this split configuration is rotated four times for full coverage of the dataset. The size of folds was selected based on the sparsity of the test data. Within the 75% of the training data, we also perform another step of cross-validation for parameter tuning of the SVM kernels. Here we apply five-fold cross-validation, where we train with 80% of the data (for a given parameter configuration) and test on the remaining 20%.

We observe in Table 2 that on average the classifiers exhibit good accuracy and F1-score. The macro-average F1-score for the ten hallmarks is 54.9%, and micro-average of 54% and accuracy of 96.3%, while the average F1-score for the entire taxonomy is approximately 52% and accuracy of 97.9%. The classifiers perform well when considering the inter-annotator agreement (κ = 0.67 for the ten hallmarks, and κ = 0.61 for the entire taxonomy), as well as the fact that on average, about 10% of the sentences in the corpus are labelled with a hallmark.

The performance is lower for some of the leaf subclasses of the taxonomy (for example, 8.1 Immune response). This is because of the low number of positive examples associated with these subclasses in the annotated corpus, and therefore, the set of discriminating features extracted by our pipeline is sparse.

We also evaluated the usefulness of our features using leave-one-out feature analysis, where each of the seven feature types is removed from the full feature set. The decrease in performance (if any) resulting from the removal of a feature type indicates its proportional positive contribution to the classification process. We use an identical experimental setup as previously.

We summarize the results of leave-one-out feature analysis in the Supplementary Material (Supplementary Table S2). Overall, the results of the analysis is consistent with a similar analysis by Baker *et al.* (2016b). The results show that the most influential feature type is lemmatized bag of words (LBoW), followed closely by the semantic distance feature (SD), where both lead to a significant decrease in performance when removed. Verb clustering (VC) was the weakest feature type; on average, it resulted in a marginal performance improvement when removed; however, it was still a useful feature for many hallmark classes.

### 3.2 Case studies

To evaluate the practical usefulness of CHAT for cancer research, we present here four example case studies. Our aim is to test whether CHAT can classify the broad and varied range of text accurately into the relevant classes of the HoC taxonomy. The results for each case study are described below and are illustrated in Figure 6.

#### 3.2.1 Case study 1: Lung cancer and cisplatin

We used CHAT to analyze PubMed literature on lung cancer and the commonly used drug to treat this cancer, cisplatin (Fig. 6A). Cell invasion and metastasis is the most common hallmark associated with lung cancer in the classified literature, which is in line with existing knowledge (Nguyen *et al.*, 2009). Cisplatin interferes with DNA replication, which kills cells through apoptosis (Wang and Lippard, 2005). Our automatic literature analysis, showing apoptosis as the most frequent hallmark associated with cisplatin, demonstrates the ability of the tool to efficiently and accurately classify the literature. Furthermore, cisplatin studies have a hallmark profile more similar to that of lung cancer than that of colorectal cancer. This might reflect the more common use of cisplatin in lung cancer treatment.

#### 3.2.2 Case study 2: Aspirin and colorectal cancer

Low-dose aspirin treatment is used to prevent colorectal cancer. As for lung cancer, the automatic literature analysis on colorectal cancer shows that invasion and metastasis is the most common cancer hallmark in the classified literature (Fig. 6B). The literature profile of aspirin shows inflammation as the most common cancer hallmark associated with aspirin, which is in line with the fact that targeting inflammation is one of the key mechanisms by which aspirin acts to prevent colorectal cancer (Drew *et al.*, 2016).

#### 3.2.3 Case study 3: Growth factor EGF and VEGF

Epidermal growth factor (EGF) and vascular endothelial growth factor (VEGF) are important in human cancers. EGF stimulates cell proliferation by binding to its receptor EGFR (Normanno *et al.*, 2006), whereas VEGF and its cognate receptor play a central role in angiogenesis (Zhao and Adjei, 2015). The CHAT classification shows that sustaining proliferative signalling and angiogenesis are the most common hallmarks associated with EGF and VEGF, respectively, in literature (Fig. 6C).

#### 3.2.4 Case study 4: Housekeeping genes TBP and GAPDH

Housekeeping genes (HKG) are often used as reference genes when studying alterations in gene expression as a response for instance, to cellular stresses (Iyer *et al.*, 2017). HKGs are expected to maintain constant expression levels in different conditions. Here we have analyzed two HKGs i.e. TATA-Box binding protein (TBP) and Glyceraldehyde 3-phosphatase dehydrogenase (GAPDH). The CHAT classification shows that the classical GAPDH significantly associated with cellular energetics and Warburg effect, while TBP does not show any significant association with any of the hallmarks (Fig. 6D). This data are in line with the experimental findings showing HKGs may be affected and respond differently depending on stress conditions (Iyer *et al.*, 2017).

## 4 Discussion

Comprehensive and efficient use of existing scientific knowledge is critically important for generating novel ideas for cancer research. Scientists working in this area use systems such as PubMed to gather existing information of relevance to their research. However, given the wide range and complexity of cancer-related scientific data and the number of relevant keywords, their synonyms and potential combinations exceeds what a scientist can reasonably memorise and handle. A dedicated tool capable of identifying and semantically organizing cancer-related scientific literature in meaningful categories is required for thorough review of literature and identification of the molecular processes involved in cancer development. The novel tool we have introduced in this paper is specifically aimed at filling this need. CHAT analyses and classifies cancer-related literature based on the widely used HoC framework (Hanahan and Weinberg, 2011). The tool’s interface, designed in collaboration with cancer researchers, enables users to immediately analyze the correlation between any query term and the hallmarks and the associated process according to the detailed HoC taxonomy introduced in this paper. Furthermore, the tool provides a variety of statistical analyses and visualizations of the hallmark annotations in their original sentence context.

Our earlier paper reported the first attempt to classify text according to the ten HoC by abstract (Baker *et al.*, 2016b). CHAT performs much finer-grained classification according to the HoC taxonomy and also at the level of sentence. Sentence-level classification allows us to capture co-occurrences between the search query and the classified hallmark at a more granular text window, thereby extracting less noisy correlations. However, in comparison with abstract-level (or document-level) classification, sentence-level classification is a more difficult NLP problem. The much smaller context window available as input to the classifier tends to reduce the classifier’s accuracy. However, this reduction in accuracy is a good trade-off compared to the gains we achieve by using a large classification window. This is evidently true as less than 10% of the sentences are associated with any hallmarks in the annotated data, i.e. most sentences will not contain any hallmark-related information, and therefore standard co-occurrence measurements such as PMI would be too noisy if used with abstract-level classification.

An important part of the tool development was refining the original ten HoC by further extending them with twenty-seven subclasses, representing the most important cellular processes involved in cancer development and progression, as described in (Hanahan and Weinberg, 2011). We also developed an improved approach for the sentence-level classification which utilizes a new set of features and NLP tools, and a new sentence-level annotated corpus. We make all these resources available under open licenses.

We showed that the NLP pipeline performed with promising accuracy, particularly given the challenges of sentence-level classification. Our case studies focused on cancer types, therapeutics, growth promoting proteins and housekeeping genes, showed that CHAT identifies correlations that agree with existing knowledge on cancer types, therapeutics and housekeeping genes. The tool proved useful for classifying cancer-related text and text mining associated biological processes, with a simple search query on cancer types, intrinsic or extrinsic factors, and therapeutics.

In future, the tool could be improved in different ways, for instance to distinguish between positive and negative evidence for a particular hallmark or to distinguish between reported facts and speculations. Also the literature search functionality can be extended to access other relevant literature databases. In addition, the classification can be refined to consider journal impact factors, citation frequencies, and cross references, which would help cancer researchers to identify, for instance more prominent, less important and incremental published data, as well as studies forming clusters. The tool can also be extended to support time-trend analysis of the scientific data related to cancer.

## 5 Conclusions

We introduced here a novel text mining tool: CHAT, capable of analyzing and classifying text on a large-scale using the publicly available abstracts of 2016 PubMed baseline (over 24 million abstracts, and over 150 million sentences), according to the evidence they provide for the Hallmarks of Cancer (HoC) and associated processes (Hanahan and Weinberg, 2011).

We evaluated CHAT intrinsically using and have demonstrated a reliable level of accuracy. We also demonstrated the usefulness of CHAT in four case studies, where we compare the hallmark prediction of CHAT of different drugs, cancers, genes and growth factors, which has been consistent with established facts in nature.

The ability of CHAT to semantically organize literature according to the hallmarks can support both basic and applied research, for instance cancer drug development, biomarker discovery and identification of previously unknown associations between genes, proteins, signalling networks, tumour types, drug, chemicals and other entities. This, in turn, may help and reduce the disease burden through preventive, diagnostic and therapeutic strategies.

## Funding

This work was supported by the Commonwealth Scholarship Commission and the Cambridge Trust (to S.B.), by Vinnova (to I.S.) and by MRC grant MR/M013049/1.

*Conflict of Interest*: none declared.

## Supplementary Material

Supplementary Data
