# Supplementary material for: Cancer Hallmarks Analytics Tool (CHAT): a text mining approach to organize and evaluate scientific literature on cancer
Source: Bioinformatics. 2017 Jul 14;33(24):3973–81. doi: 10.1093/bioinformatics/btx454 (PMC5860084; doi:10.1093/bioinformatics/btx454)
Supplement: Supplementary Data [file btx454_supplementary.pdf]

Supplementary Material

Table S1. Number of features per feature type for each hallmark classifier after feature selection.

Hallmark	LBoW	Bigram	Trigram	VC	NE	MeSH	Chem	SD	Total
**1. Sustaining proliferative signalling**	2576	1167	239	122	390	280	129	2576	4903
1.1 Cell cycle	1359	423	68	112	144	123	43	1359	2272
1.2 Growth factors growth promoting signals	1270	357	51	111	159	134	55	1270	2137
1.2.1 Downstream signalling	740	176	19	91	82	75	29	740	1212
1.3 Receptors	1338	397	54	111	158	118	44	1338	2220
**2. Evading growth suppressors**	1588	508	78	120	147	154	54	1588	2649
2.1 By deregulating cell cycle checkpoints	1106	304	50	106	101	101	34	1106	1802
2.1.1 Cell cycle	1047	289	44	105	96	91	28	1047	1700
2.1 By evading contact inhibition	765	116	13	104	49	45	7	765	1099
**3. Resisting cell death**	2551	1035	215	119	295	261	114	2551	4590
3.1 Apoptosis	2081	837	173	114	246	216	93	2081	3760
3.2 Autophagy	441	73	5	64	33	30	11	441	657
3.3 Necrosis	733	66	2	89	25	31	3	733	949
**4. Enabling replicative immortality**	931	172	11	105	67	80	26	931	1392
4.1 Immortalization	484	54	2	91	35	35	8	484	709
4.2 Senescence	647	109	5	94	48	53	17	647	973
**5. Inducing angiogenesis**	1104	266	46	104	105	97	28	1104	1750
5.1 By deregulating angiogenesis	1079	253	43	103	104	91	24	1079	1697
5.1.1 Angiogenic factors	669	119	20	88	66	51	14	669	1027
**6. Activating invasion and metastasis**	1922	718	114	115	212	172	43	1922	3296
6.1 Invasion	1209	365	50	103	141	100	25	1209	1993
6.2 Metastasis	1312	369	55	111	105	98	21	1312	2071
**7. Genomic instability and mutation**	2179	652	95	127	150	216	77	2179	3496
7.1 DNA damage	1414	320	40	118	76	107	33	1414	2108
7.1.1 Adducts	397	18	1	75	10	16	4	397	521
7.1.2 Strand breaks	633	87	5	93	32	27	5	633	882
7.2 DNA repair mechanisms	1008	174	22	105	51	84	31	1008	1475
7.3 Mutation	857	96	3	108	41	65	15	857	1185
**8. Tumour promoting inflammation**	1808	520	64	115	157	147	40	1808	2851
8.1 Immune response	422	42	2	79	22	25	3	422	595
8.2 Inflammation	1730	488	58	113	146	141	39	1730	2715
8.2.2 Oxidative stress	1054	213	20	98	69	74	23	1054	1551
**9. Cellular energetics**	797	164	17	100	43	68	20	797	1209
9.1 Glycolysis/Warburg effect	744	153	15	97	39	59	19	744	1126
**10. Avoiding immune destruction**	900	162	18	102	73	68	14	900	1337
10.1 Immune response	690	103	9	89	57	50	8	690	1006
10.2 Immunosuppression	429	44	2	82	21	25	3	429	606
**Average:**	1136	308	47	102	103	98	32	1136	1825

Table S2. Leave-one-out feature analysis results. All figures are F1-Scores (%).

Hallmark	All	LBoW	n-gram	VC	NE	MeSH	Chem	SD
**1. Sustaining proliferative signalling**	47.3	40.6	42.4	47.6	46.7	45.7	47.8	36.4
1.1 Cell cycle	53.8	39.2	50.2	53.4	54.5	49.9	54.0	40.9
1.2 Growth factors growth promoting signals	30.6	26.0	28.7	31.1	29.2	30.8	31.1	23.5
1.2.1 Downstream signalling	34.0	23.8	29.4	33.9	32.0	35.7	33.3	26.6
1.3 Receptors	41.4	29.7	40.6	42.3	41.1	40.5	41.4	33.9
**2. Evading growth suppressors**	47.9	39.3	45.1	49.7	48.5	45.4	50.2	39.7
2.1 By deregulating cell cycle checkpoints	39.5	30.9	34.7	39.6	39.6	40.1	42.4	30.8
2.1.1 Cell cycle	39.1	30.6	36.7	40.0	39.5	38.8	40.7	33.6
2.1 By evading contact inhibition	75.1	72.4	65.2	76.9	74.3	75.6	75.2	72.6
**3. Resisting cell death**	66.9	44.8	66.3	66.9	66.3	69.5	67.1	50.2
3.1 Apoptosis	69.0	48.9	63.7	70.3	69.3	60.5	69.8	53.1
3.2 Autophagy	69.1	49.2	66.9	68.8	69.8	70.5	68.7	52.5
3.3 Necrosis	71.6	50.9	68.9	74.2	70.7	64.7	71.7	52.0
**4. Enabling replicative immortality**	69.9	42.1	60.1	69.2	69.0	69.4	68.2	57.3
4.1 Immortalization	67.2	30.0	65.0	67.7	65.3	67.2	65.0	55.1
4.2 Senescence	72.6	63.9	68.2	71.9	72.7	66.1	72.2	65.3
**5. Inducing angiogenesis**	50.0	38.3	51.7	49.3	50.3	50.3	50.4	40.0
5.1 By deregulating angiogenesis	49.9	38.9	50.2	49.4	50.2	50.5	50.2	39.9
5.1.1 Angiogenic factors	47.3	36.8	48.0	44.6	46.8	45.9	48.2	36.4
**6. Activating invasion and metastasis**	63.4	39.9	64.2	62.9	63.7	62.0	63.8	51.4
6.1 Invasion	55.6	34.4	51.7	56.4	55.6	58.4	58.2	47.8
6.2 Metastasis	61.3	29.6	61.4	61.9	61.3	63.0	60.3	53.4
**7. Genomic instability and mutation**	48.4	36.8	46.8	48.4	48.0	50.2	48.4	38.2
7.1 DNA damage	50.5	36.7	48.2	50.0	49.3	50.1	50.3	39.9
7.1.1 Adducts	61.0	29.7	58.5	62.9	57.4	60.8	57.1	51.2
7.1.2 Strand breaks	38.8	20.9	35.5	35.6	36.4	38.5	40.0	34.9
7.2 DNA repair mechanisms	47.7	31.3	41.2	47.3	47.1	46.9	49.0	41.0
7.3 Mutation	46.0	24.8	44.0	46.8	46.0	44.5	45.5	40.9
**8. Tumor promoting inflammation**	50.1	34.0	50.1	50.6	49.9	51.3	51.2	38.6
8.1 Immune response	29.0	10.7	24.1	30.1	27.9	22.2	27.3	24.1
8.2 Inflammation	51.8	35.1	51.6	52.8	52.2	47.1	52.8	43.0
8.2.2 Oxidative stress	52.7	40.2	53.7	54.8	52.0	54.9	54.2	43.7
**9. Cellular energetics**	58.2	41.0	55.4	57.2	57.7	60.9	57.7	51.8
9.1 Glycolysis/Warburg effect	57.8	40.4	53.8	57.8	57.0	51.9	58.1	49.7
**10. Avoiding immune destruction**	41.7	23.6	42.1	44.0	43.8	41.8	44.7	33.4
10.1 Immune response	28.9	20.3	28.3	33.5	30.7	27.1	33.8	25.1
10.2 Immunosuppression	50.7	42.5	48.0	55.4	52.5	48.3	52.2	43.6
**Average:**	52.3	36.4	49.7	52.8	52.0	51.3	52.8	43.0
